# Influential Factors on Intracanal Post Selection for Endodontically Treated Teeth in Kurdistan Province of Iran: Influential Factors on Intracanal Post Selection

**DOI:** 10.31661/gmj.v13iSP1.3619

**Published:** 2024-12-08

**Authors:** Payam Younesi Baneh, Elaahe Kebriyaei, Shiva Mahboubi

**Affiliations:** ^1^ Student Research Committee, Kurdistan University of Medical Sciences, Sanandaj, Iran; ^2^ Faculty of Dentistry, Kurdistan University of Medical Sciences, Sanandaj, Iran; ^3^ Department of Prosthodontics, Faculty of Dentistry, Kurdistan University of Medical Sciences, Sanandaj, Iran

**Keywords:** Post System, Endodontically Treated Teeth, Dentist

## Abstract

**Background:**

Due to severe coronal destruction, endodontically treated teeth (ETT) often require intracanal posts to provide retention for the restorative material. This study aimed to evaluate the opinions and practices of Iranian dentists regarding the selection of intracanal posts for ETT reconstruction.

**Materials and Methods:**

This cross-sectional study was conducted among dentists in Kurdistan province, a region of west of Iran,, who have experience in performing ETT reconstruction. A questionnaire was designed, comprising two parts: the first part collected demographic information. The second part assessed the criteria for selecting post systems for ETT, including 12 questions that inquired about the placement of intracanal posts, indications for post placement, post material preferences, post length and diameter, and fabrication methods. Collected data were coded and analyzed using SPSS version 20 (IBM Corp., Armonk, NY, USA).

**Results:**

Totally, 96.6% of the participants used posts for ETT. They believed that not all ETT require a post. Also, 53.8% believed that posts do not reinforce ETT; 83.8% mentioned the remaining tooth structure to be the main criterion in choosing between prefabricated and cast posts and cores. Cast posts and cores were used in 62.4% of anterior and 56.4% of posterior teeth. Prefabricated metal posts were used by 68.3% of dentists. Also, 51.3% used base metal alloys for the cast posts and cores; 44.4% stated that at least 4 mm of gutta-percha must remain at the apex, and 45.3% stated that the post diameter should be one-third of the root diameter.

**Conclusion:**

The study found that Iranian dentists have varying opinions and practices regarding the selection of intracanal posts for endodontically treated teeth reconstruction, with a preference for cast posts and cores and base metal alloys, and consideration of remaining tooth structure as the main criterion for post selection.

## Introduction

Endodontic treatment is an integral dental procedure [1]. Reduction in fracture resistance of endodontically treated teeth (ETT), mainly due to insufficient residual coronal structure, is a serious problem with adverse clinical complications [2]. The main goal of endodontic treatment is to save the tooth. The success of the treatment largely depends on the quality of the final restoration, rather than the endodontic treatment itself. A poor restoration can lead to tooth fracture and ultimately, extraction [3]. A proper coronal restoration should restore tooth function and appearance, and prevent bacterial leakage into the root canal system [4].

Different types of materials and techniques are used for restoration of ETT, ranging from relatively small direct restorations to more complex indirect restorations that require placement of an intracanal post [5]. Intracanal posts are not intended to increase the fracture resistance of teeth [6][7]; instead, they are placed to ensure optimal retention for the core material [7][8][9]. The decision regarding placement of intracanal posts should be made based on the position of tooth in dental arch [7][10], the amount of remaining coronal structure [9][11], and the functional requirements of the tooth (for example, as a retainer for removable or fixed restorations) [12]. Post systems to retain restorative materials fall into two categories: prefabricated posts (made from materials like stainless steel, gold, titanium, or composite resin) and cast (customized) posts and cores [2]. Metal posts come in various designs; their length and design, not diameter, affect retention. Longer posts are preferred, but 4-5mm of gutta-percha should be preserved for an optimal seal [11].

The treatment options for each tooth are influenced by several factors, and choosing the type of post system for tooth reconstruction is a difficult decision for many dental clinicians. Most dentists reconstruct ETT mainly based on their own previous experiences irrespective of the most recent scientific data and principles [13]. Also, dentists’ treatment choices are influenced by their personal preferences, professional experience, and geographical region [14]. Many studies have been conducted on the methods of choosing the post system for ETT by dentists in different regions [7][9][14][15][16], but no such a study is available on Iranian dentists. Therefore, this study aimed to investigate the attitudes and preferences of Iranian dentists towards the selection of intracanal posts for ETT reconstruction.

## Materials and Methods

This cross-sectional study was done between dentists who working in Kurdistan province, a region of West of Iran, from March to November 2021. The study protocol was approved by university intuitional review board with ethics code of IR.MUK.REC.1398.283. Participants were selected from a WhatsApp group of dentists in Kurdistan, which had 271 members. All members of the group were invited to participate in the study through a mass message, and those who were willing to participate were asked to complete a questionnaire.

The inclusion criteria included dentists who have experience of performing ETT reconstruction and were willing to participate in the study who were enrolled after signing informed consent forms. No specific exclusion criteria were employed in this study.

A questionnaire was designed [3][5][7][9][13][14][16]. The first part of the questionnaire included 6 questions regarding the demographic information of dentists (age, gender, academic degree, workplace, participation in continuing education courses, and congresses, and dental work experience). The second part included 12 questions regarding the criteria for selecting the post system for ETT by dentists. These questions inquiry about their preference for using intracanal posts, whether they believe it reinforces ETT and reduces fracture risk, and the criteria for choosing between prefabricated and cast posts. The survey also asks about the type of post used for anterior and posterior ETT, preferred materials and designs for prefabricated posts, and alloys for cast posts. Additionally, it seeks opinions on post length and diameter, as well as the method used for creating cast posts and cores.

To ensure the validity and reliability of the questionnaire, a Content Validity Index (CVI) and Content Validity Ratio (CVR) analysis were performed by a panel of 10 experts in the field of endodontics and dental education. The experts were asked to evaluate the relevance, clarity, and simplicity of each item, and their feedback was used to calculate the CVI and CVR values. The results showed that all items had satisfactory CVI and CVR values, ranging from 0.8 to 1.00, indicating excellent content validity.

After collecting the questionnaires, they were coded. Statistical analysis was performed using SPSS version 20 (IBM Corp., Armonk, NY, USA) anonymously, and frequencies/percentages, mean and Standard Deviation (SD) were used for descriptive statistics.

## Results

**Table:** Table1. Demographic Variables Included: 1. Gender 2. Academic Degree 3. Workplace 4. Participation in Continuing Education Courses and Congresses 5. Dental Work Experience (N=117)

**Demographic variable**	**Frequency (n)**	**Percentage (%)**
**Gender**		
Male	88	75.2
Female	29	24.8
**Academic degree**		
General dentist	103	88
Dental specialist	14	12
**Workplace**		
Private office	65	55.6
Private office and dental clinic	52	44.4
**Participation in counting education courses and congresses **		
Yes	97	82.9
No	20	17.1
**Dental work experience**		
< 6 years	34	29.1
6-10 years	26	22.2
10-16 years	1	16.2
16-20 years	12	10.3
> 20 years	25	21.1
Retired	1	0.9

**Table:** Table2. The Questionnaire used for Data Collection and the Frequencies and Percentages of Them

**Question**	**Frequency (n)**	**Percentage (%)**
**Do you use intracanal posts for ETT?**		
Yes	113	96.6
No	4	3.4
**Do you think that all ETT require an intracanal post?**		
Yes	9	7.7
No	108	92.3
**Do you believe that intracanal posts reinforce ETT and decrease the possibility of fracture? **		
Yes	52	44.4
No	63	53.8
I do not know	2	1.7
**What is the main criterion in choosing between prefabricated posts and cast posts? **		
Remaining tooth structure	98	83.8
Ease of use	24	20.5
Reducing the number of visits	22	18.8
Ease of removal if endodontic retreatment is required.	24	20.5
Cost	34	29.1
Esthetics	9	7.7
Tooth position (anterior or posterior)	35	29.9
Canal size	17	13.8
Geometry of crown destruction	15	12.8
Others	1	0.9
**what type of post do you use more often for anterior ETT? **		
Prefabricated	44	37.6
Cast	73	62.4
**What type of post do you use more often for posterior ETT? **		
Prefabricated	66	56.4
Cast	51	43.6
**If you use a prefabricated post, which type do you mostly prefer? **		
Metal	80	68.3
Fiber	32	27.4
Ceramic	3	2.6
Others	2	1.7
**If you use prefabricated metal posts, which design do you prefer? **		
Parallel sided	16	13.7
Tapered	45	38.5
Parallel sided+ tapered	33	28.2
Screw type	23	19.7
**If you use cast posts and cores, which alloy do you prefer? **		
**Noble**	20	17.1
**Base metal**	60	51.3
**Titanium**	30	25.6
**Zirconia**	4	3.4
**Others**	3	2.6
**In your opinion, what is the appropriate post length?**		
**As long as the clinical crown**	16	13.7
**1/3 of root length**	4	3.4
**2/3 of root length**	39	33.3
**1/2 of root length**	1	0.9
**Leaving 4 mm of gutta-percha in the canal**	52	44.4
**Others**	5	4.3
**In your opinion, what is the appropriate post diameter?**		
**1/3 of root diameter**	53	45.3
**½** **of root diameter**	15	12.8
**2/3 of root diameter**	5	4.3
**It depends on the remaining tooth structure**	43	36.7
**Others**	1	0.9
**If you use cast posts and cores, which method do you mostly use? **		
**Direct method**	58	49.6
**Indirect method**	30	25.6
**Both of them**	29	24.8

Among 271 members, 247 had ever performed an ETT reconstruction that were in invited and 134 responded to the questionnaire; but only 117 records were complete for analysis (54.25% participation rate).

The mean age of the participants was 38.41±9.38 years (range 25-58 years). Other demographic variables are shown in Table-1. The majority (75.2%) were male, and most (88%) held a general dentist degree. The majority of participants worked in a private office (55.6%), with 44.4% working in a private office and dental clinic. A large proportion (82.9%) of participants had attended continuing education courses and congresses. The participants had varying levels of dental work experience, with 29.1% having less than 6 years of experience, 22.2% having 6-10 years, and 21.1% having more than 20 years of experience. Only one participant was retired.

The majority of dentists reported placement of intracanal posts in ETT (96.6%). The majority of them did not think that intracanal posts should be placed in every ETT (92.3%). Almost half of them (53.8%) believed that posts do not reinforce ETT. The majority of them referred to the remaining tooth structure as the main criterion to consider in choosing between prefabricated posts and cast posts and cores (83.8%). More than half of the dentists used cast posts and cores in the anterior ETT (62.4%). Almost half of them used prefabricated posts for the posterior ETT (56.4%) (Table-2).

More than half of the dentists used metal posts in case of using a prefabricated post (68.3%). The tapered type was the most frequently used prefabricated metal post (38.5%). Base metal alloys were most frequently used for the cast posts and cores (51.3%). Regarding the proper post length, the majority of participants reported retaining 4 mm of gutta-percha in the apical region (44.4%). The most suitable post diameter was one-third of the root diameter (45.3%). Regarding the method of fabrication of cast posts and cores, the direct method was more commonly selected (49.6%) (Table-2).

## Discussion

In the present study, opinions and practices of Iranian dentists regarding the selection of intracanal posts for ETT reconstruction were investigated. This study included 117 dental clinicians, with a mean age of 38.41 years, consisting of 88 males and 29 females, and comprising 103 general dentists and 14 dental specialists. Based on the results of the present study, the majority of participants (96.6%) reported using intracanal posts for ETT, which was almost similar to other studies [7][9]. However, this result was contrary to the findings of Brunton et al., who reported the use of intracanal posts in one-third of anterior and 15% of posterior teeth [17]. Also, Hussey et al. reported that dentists used intracanal posts in 44% of anterior and 25% of posterior teeth [18]. In a study by Rabi et al., 58.7% of dentists used intracanal posts in 30-50% of the teeth that served as a retainer [14].

Also, 92.3% of dentists in the present study believed that not all ETT require a post, which was similar to the results of previous studies [7][9][13][14][16][19]. This finding is due to the fact that less damaged teeth do not require an intracanal post for retention of final restoration [9], and dentists evaluated in this study were well aware of this fact.

A total of 44.4% of dentists believed that intracanal posts reinforce ETT, which is much lower than the rate in the study by Alenzi et al., (82.9%) [9]. It was also lower than the rate in some other studies [5][10][12][16]. In a study by Habib et al., 12% and 4% of general dentists and dental specialists believed in the reinforcing effect of intracanal posts, respectively [3]. Ahmed et al. reported a more favorable result than other studies such that 88% of dentists in their study (out of 1008 dentists living in 10 American states) correctly stated that the primary function of intracanal posts is to retain the core material and they do not play a role in reinforcing the ETT [11]). Most evidence-based studies have shown that posts do not reinforce ETT, and are not necessarily required for all ETT. Intracanal posts are only used to retain the restorative material [6][13].

The choice between prefabricated and cast posts and cores is influenced by several factors, including the extent of tooth structure loss, procedural simplicity, number of sessions, cost, esthetic considerations, and tooth position. Specifically, cast posts and cores are preferred when there is significant tooth structure loss, as they can be customized to match the remaining tooth structure [7][20]. Also, cast post and core systems better match highly conical canals, those with a non-round cross-section, and those with an irregular shape [21].

Most of the participants in the present study (83.8%) referred to the remaining tooth structure as the main criterion to consider in choosing the type of post, which is similar to the findings of Alenzi et al. that most participants (77.4%) reported that, as well as the Kon et al. [9][12].

Regarding the type of post, cast post and core (62.4%) and prefabricated posts (56.4%) were more frequently preferred for the anterior and posterior teeth, respectively. In studies by Kon et al., and Seow et al., cast posts and cores and metal posts were preferred for the anterior and posterior teeth, respectively [5][12]. In other studies, the majority of participants preferred the use of prefabricated posts over cast posts and cores [7][9][10][14][16]. The prefabricated posts are used due to their ease of use and completion of treatment in one session [9]. However, the right treatment should not be sacrificed for the ease of procedure, and the right treatment should be chosen for each tooth.

In choosing among different types of prefabricated posts, metal posts (68.4%) followed by fiber posts (27.4%) were more commonly chosen. In similar studies, dentists preferred prefabricated metal posts [12][13][18][22], but fiber posts were used by the majority of dentists in studies by Alenzi et al., Sumitha et al., and Naumann et al. [9][10][11]. Fiber posts are currently preferred considering the similarity of their elastic modulus to that of dentin, and more even stress distribution in the post and dentin. Resultantly, root fractures can be more easily reconstructed [9][23][24][25].

Regarding the design of prefabricated metal posts, tapered posts (38.5%) and parallel posts (13.7%) were the most and least popular posts, respectively. In the study by Alenzi et al., the majority of participants (59.8%) preferred tapered posts [9]. In the study by Sumitha et al., tapered posts (50.2%) were preferred to parallel posts (42.46%) [11]. In the study by Eckerbom et al., parallel posts were preferred [22]. Canal preparation for parallel posts requires special burs that can weaken the root [9]. Screw posts have a significantly shorter lifespan due to the stress applied to the root [10], which were preferred by 19.7% of dentists in the present study. In other studies, screw posts were more favored than other prefabricated metal posts [7][12][14][16]. No post system is suitable for all teeth, and the appropriate system should be chosen depending on the canal shape.

Regarding the material of cast posts and cores, most of the participants (51.3%) chose base metal alloys for casting posts, which can be due to the lower price of these alloys compared to noble alloys, titanium, and zirconia. In the study by Alenzi et al., 62.8% of dentists used cast base metal posts and cores [9], but in the study by Brunton et al., noble alloy was the most commonly used material for posts [17].

In addition to the opinion of dental clinicians, the choice of alloy type in cast post and core systems can also be influenced by the financial status of patients.

Regarding the appropriate post length, the majority of participants (44.4%) reported retaining 4 mm of gutta-percha at the end of the canal. In the study by Rabi et al., most dentists (36.6%) stated the post length to be one-third of the root length [14].

In the study by Kon et al., more than 60% of dentists believed that the post length should be two-thirds of the root length to gain canal anchorage [12] and in other studies, most of the participants chose the post length to be two-thirds of the root length or retaining 3-4 mm of gutta-percha in the apical region [5][13][16][18].

Variations in the reported results can be due to differences among different populations as well as different trainings using different references. Regarding the appropriate post diameter, the majority of respondents (45.3%) chose one-third of the root diameter. It is never recommended to increase the canal diameter to increase the post diameter with the aim of increasing the retention, because the retention increases only slightly while the root is weakened at the same time. Experimental evidence shows that if the post diameter is not more than one-third of the root cross-section, the treatment prognosis will be good [26][27].

The direct method (49.6%) was mostly chosen for the fabrication of cast posts and cores. The studies that compared direct and indirect methods stated easier fabrication and better marginal adaptation of intracanal posts in the indirect method, compared with the direct method [28].

Depending on the access to the tooth, the direct technique is more appropriate for single-canal teeth with good access while the indirect method may be preferred for multiple canals or teeth with difficult access [27]. However, the indirect method has become very popular with the use of intraoral scanners.

The main limitation of the present study was small number of dental specialists in the study population, which made it impossible to compare general dentists and specialists in terms of the criteria for selecting the post system. Therefore, similar studies with an equal population of general dentists and specialists are recommended to compare them in this respect. This study relied on self-reported data from dentists, which may not accurately reflect their actual clinical practices. Furthermore, the study did not assess the experiences of dentists with the outcomes of their work, such as the success rates of post placements or the incidence of post-related complications.

## Conclusion

Despite the limitations of this study, the following results were obtained:

The majority of dentists were aware of the fact that not all ETT require an intracanal post and referred to the remaining tooth structure as the main criterion to consider in choosing the type of post (prefabricated or cast). They mostly used cast posts and cores for the anterior teeth and prefabricated posts for the posterior teeth. Among the prefabricated posts, most dentists used the metal type followed by fiber posts.

Tapered posts were the most popular post design in prefabricated metal posts in the present study. Base metal alloys were the most popular material for the fabrication of cast posts and cores. Regarding the most suitable length of the post, the majority of dentists recommended retaining 4 mm of gutta-percha at the end of the canal. The most suitable post diameter was one-third of the root diameter and regarding the cast post and core fabrication method, the majority of dentists preferred the direct method.

## Conflict of Interest

The authors declare no competing interests.
